# Transcriptome Analysis of the Harmful Dinoflagellate *Heterocapsa bohaiensis* Under Varied Nutrient Stress Conditions

**DOI:** 10.3390/microorganisms12122665

**Published:** 2024-12-22

**Authors:** Peng Peng, Fangxin Han, Xue Gong, Xiangyuan Guo, Ying Su, Yiwen Zhang, Jingjing Zhan

**Affiliations:** 1 School of Chemical Engineering, Ocean and Life Sciences, Dalian University of Technology, NO.2 Dagong Road, Panjin 124221, China; dn823237388@163.com (P.P.); m18230283739@outlook.com (X.G.); m18230283739@163.com (X.G.); yingsu@dlut.edu.cn (Y.S.); jingjingzhan@dlut.edu.cn (J.Z.); 2 School of General Education, Dalian University of Technology, NO.2 Dagong Road, Panjin 124221, China; hanfangxin@dlut.edu.cn

**Keywords:** *Heterocapsa bohaiensis*, transcriptomics, metabolic mechanisms, phosphorus limitation, nitrogen limitation, gene expression, cultivated

## Abstract

The increasing prevalence of harmful algal blooms (HABs) driven by eutrophication, particularly in China’s nearshore waters, is a growing concern. Dinoflagellate *Heterocapsa bohaiensis* blooms have caused significant ecological and economic damage, as well as mass mortality, in cultivated species. Nutrients are one of the primary inducers of *H. bohaiensis* blooms. However, the transcriptomic studies of *H. bohaiensis* remain sparse, and its metabolic pathways are unknown. This study analyzed the transcriptome of *H. bohaiensis* under varying nutrient conditions (nitrogen at 128, 512, and 880 μM; phosphate at 8, 6, and 32 μM), focusing on differential gene expression. The results indicated that deviations in nutrient conditions (higher or lower N:P ratios) led to a higher number of differentially expressed genes compared to the control (N:P ratios = 27.5), thereby underscoring their pivotal role in growth. Gene Ontology (GO) enrichment analyses showed that nutrient limitation upregulated the biosynthesis and catabolism processes while downregulating the cell cycle and division functions. The Kyoto Encyclopedia of Genes and Genomes (KEGG) analysis revealed that, under nitrogen limitation, the proteasome pathways were upregulated, while photosynthesis and carbon fixation were downregulated; under phosphorus limitation, the proteasome pathways were upregulated and nitrogen metabolism was downregulated. These findings suggest that *H. bohaiensis* adapts to nutrient stress by adjusting its metabolic processes.

## 1. Introduction

Harmful algal blooms (HABs) have become one of the major environmental issues of global concern, causing serious impacts on the marine environment, aquaculture, and the offshore economy [1,2]. Since the 1980s, the reports of HABs in China’s nearshore waters have increased exponentially on a decadal scale [3,4]. Due to the continuous changes in the global climate and marine environment, as well as human activities, the severity of HABs in China’s offshore waters has gradually increased, and new HAB species are likely to appear in the future. Dinoflagellates are responsible for most marine HABs and are one of the most harmful algae to aquaculture [5,6]. The factors affecting the formation of HABs include eutrophication (nitrogen, phosphorus, silica, and nutrient ratios, among others), changing climate, biological factors, and other various factors that have synergistic effects on HABs [7,8]. Studies have shown that elevated nutrient loading from agricultural runoff and wastewater discharge could lead to increased frequency and intensity of HABs [9]. In parallel, the impact of climate change, including rising sea temperatures, altered precipitation patterns, and increased frequency of extreme weather events, further compounds the effects of eutrophication. Warmer water temperatures could enhance algal growth rates and metabolic activity, thus facilitating the formation of blooms [10]. Adequate and appropriate proportions of nutrients such as nitrogen and phosphorus are the basis for the occurrence of HABs; different species groups display preferences for specific nutrient ratios [11]. Previous studies have revealed that combined phosphorus and nitrogen enrichment stimulated HABs more than P or N alone [9], and different N:P ratios have sometimes been associated with physiological changes of certain harmful dinoflagellate species [12,13,14].

*Heterocapsa* spp. are bloom-forming dinoflagellates [15], such as *Heterocapsa circularisquama*, which is widely distributed worldwide and can form HABs and kill bivalves [16,17]. *Heterocapsa bohaiensis* is a newly discovered alga that has caused HABs and mass mortality regarding river crab larvae in culture ponds near Liaodong Bay in the Bohai Sea in recent years [18]. Previous studies found that hemolytic toxins were considered to be involved in the toxic mechanism of *H. bohaiensis* against rotifer *Brachionus plicatilis* [19] and achieved a competitive advantage via exploitation competition [20]. The concentrations of calcium were quantified in the mastax, stomach, and ovaries of *B. plicatilis*. It was evident that the fluorescence intensity had increased in the stomach, which indicated that calcium homeostasis and membrane permeability had been altered following the ingestion of *H. bohaiensis*. However, transcriptomic analyses of *H. bohaiensis* remain limited, and its underlying metabolic pathways have yet to be elucidated. This gap in the literature represents a significant obstacle to a comprehensive understanding of the molecular biology of *H. bohaiensis* and its ecological impacts. In this study, in order to further investigate the molecular mechanism of nitrogen and phosphorus uptake and utilization by *H. bohaiensis*, transcriptome sequencing was performed on *H. bohaiensis* cultured under different nitrogen and phosphorus concentrations. Normal f/2 medium conditions were used as a control to identify the pathways affecting the physiological activity of *H. bohaiensis* in order to elucidate the key metabolic and biological pathways regulating physiological activity, and to investigate the molecular response of *H. bohaiensis* under nitrogen- and phosphorus-limiting conditions at the RNA level. These findings may provide theoretical support for the prevention and control of *H. bohaiensis* blooms, facilitate the survival and reproduction of other aquatic organisms, promote the restoration of biodiversity in the ecosystem, and reduce the toxic effects of these blooms on aquaculture species, thereby improving the economic benefits of aquaculture.

## 2. Materials and Methods

### 2.1. Test Species and Culture Conditions

Strains of *H. bohaiensis* were obtained from Guanghe Crab Industry Limited Company from coastal aquaculture ponds of Liaodong Bay in April 2016. The strain was further enriched, isolated, and purified to sterile conditions in the laboratory of Dalian University of Technology.

*Heterocapsa bohaiensis* was cultured in an illumination incubator (Ningbo southeast instrument Co., Ltd., Ningbo, China) using 1.5 L f/2 medium [21], with three replicates in each treatment. The seawater utilized for the preparation of the medium exhibited a salinity of 27 ± 1. It was filtered through a 0.45 μm acetate membrane and subsequently subjected to autoclaving at 121 °C for 20 min. Artificial white cold light source was used as the illumination source. The illumination was about 4000 Lx, the alternating period of light and dark was set to 12 h, and the temperature was 20 ± 1 °C. These conditions were selected to replicate the natural environmental conditions under which *H. bohaiensis* typically thrives, and to enhance the applicability of our findings to natural settings, such as aquaculture ponds and coastal ecosystems. All cultures were shaken at a fixed time each day to prevent cells from lacking oxygen and adhesion. The microalgal cells were cultured in f/2 medium with five different initial N:P ratios. The experiments were conducted for 42 days. On day 27, samples were taken for transcriptome sequencing analysis as the algal cells were in the stable phase with a maximum algal density (Figure S1). The nutrient concentrations in the medium are indicated in Table 1. The remaining reagents were configured according to f/2 medium specifications. Cells were first grown in a nutrient-replete medium to ensure healthy and consistent initial growth. The nutrient concentrations were then gradually adjusted over a period of 10–15 days to the desired N:P ratios for each experimental condition. This step minimized shock responses and allowed the cells to acclimate to the new nutrient environment.

### 2.2. RNA Sequencing and Data Analysis

#### 2.2.1. RNA Extraction

*Heterocapsa bohaiensis* cells were collected by centrifugation at 4000 r min^−1^ at −4 °C for 10 min, immediately frozen in liquid nitrogen, and stored at −80 °C until RNA was extracted [22]. Total RNA was extracted from frozen cells using the Tiangen polysaccharide polyphenol kit (QIAGEN, Hilden, Germany) according to the manufacturer’s instructions. The integrity and total amount of RNA were taken as the main reference for quality control. The RNA integrity index of the samples was above 7.0, indicating high RNA quality.

#### 2.2.2. Library Preparation and Sequencing

Total RNA was used as input material for the RNA sample preparations. Briefly, mRNA was purified from total RNA by using poly-T oligo-attached magnetic beads. Fragmentation was carried out using divalent cations under elevated temperature in First Strand Synthesis Reaction Buffer(5X). First-strand cDNA was synthesized using random hexamer primer and M-MuLV Reverse Transcriptase, then use RNaseH to degrade the RNA. Second-strand cDNA synthesis was subsequently performed using DNA polymerase I and dNTP. Remaining overhangs were converted into blunt ends via exonuclease/polymerase activities. After adenylation of 3′ ends of DNA fragments, adapters with hairpin loop structure were ligated to prepare for hybridization. In order to select cDNA fragments of preferentially 370–420 bp in length, the library fragments were purified with AMPure XP system (Beckman Coulter, Beverly, NJ, USA). Then, following PCR amplification, the PCR product was purified by AMPure XP beads, and the library was finally obtained.

The library was initially quantified by Qubit2.0 Fluorometer, then diluted to 1.5 ng/μL, and the insert size of the library is detected by Agilent 2100 bioanalyzer (Santa Clara, CA, USA). After insert size meets the expectation, qRT-PCR is used to accurately quantify the effective concentration of the library (the effective concentration of the library is higher than 2 nM) to ensure the quality of the library.

After the library is qualified, the different libraries are pooled according to the effective concentration and the target amount of data off the machine and then sequenced by the Illumina NovaSeq 6000 (Novocean Corporation, Beijing, China). The end reading of 150 bp pairing is generated. Four fluorescently labeled dNTPs, DNA polymerase, and splice primers were added to the sequenced flow cell and amplified. The sequencer captures the fluorescence signal and converts the optical signal into the sequencing peak by computer software so as to obtain the sequence information of the fragment to be tested.

#### 2.2.3. Transcriptome Assembly

In order to ensure the quality and reliability of data analyses, clean data (clean reads) were obtained by removing reads containing adapter, reads containing N base, and low-quality reads from raw data. Then, the Trinity software (v2.6.6) was used to assemble the clean reads for the reference sequence obtained after the continued analysis. The assembled single genes were annotated using seven available databases: KEGG (v2.0.12), GO (1.10.0), NR (https://www.ncbi.nlm.nih.gov/protein/, 16 October 2021), NT (https://www.ncbi.nlm.nih.gov/nucleotide/, 16 October 2021), Swiss-Prot (https://proteininformationresource.org/, 18 October 2021), Pfam (v3.3.2), and KOG(https://mycocosm.jgi.doe.gov/help/kogsearch.jsf, 20 October 2021). This multi-database approach enabled a comprehensive assessment of gene function and metabolic pathways, particularly for non-model organisms such as *H. bohaiensis*. By comparing results across these diverse databases, we could elucidate the principal biological functions and metabolic pathways associated with each gene fragment in *H. bohaiensis*. The integration of these resources not only enhanced the accuracy of functional annotations but also provided a broader context for understanding the organism’s biology.

#### 2.2.4. Analysis of Differentially Expressed Genes (DEGs)

The transcriptome obtained by Trinity splicing is used as reference sequence (Ref). The BowtiE2 program in the RSEM software (v1.2.15) compared clean-read reference sequences for each sample [23]. Gene expression levels were counted according to FPKM (number of fragments per thousand bases per million mapping readings of exon model). Prior to differential gene expression analysis, for each sequenced library, the read counts were adjusted by edgeR program package through one scaling normalized factor. Differential expression analysis of two conditions was performed using the edgeR package (3.24.3). The *p* values were adjusted using the Benjamini and Hochberg method. *p*_adj_ < 0.005 and |log2 (foldchange)| >1 was set as the threshold for significantly differential expression. The GO enrichment analysis method was GOseq (1.10.0) [24], which is based on Wallenius non-central hyper-geometric distribution; *p*_adj_ less than 0.05 is significantly enriched. KOBAS (v2.0.12) software was used for KEGG pathway enrichment analysis of differential gene sets [25], again with *p*_adj_ less than 0.05 as significant enrichment. Three parallel samples were set up in each group.

## 3. Results

### 3.1. Transcriptome Assembly and Annotation

The GC content of the five samples sequenced in the experiment ranged from 60% to 65%, and the proportion of Q30 bases in each sample was greater than 92% (Table 2). A total of 95,863 unigenes were sequenced, with an average length of 1286 and good assembly quality. A total of 29,437 unigene lengths were found to be less than 500 base pairs (bp), 32,892 unigene lengths were between 500 and 1000 bp, 3097 unigene lengths were between 1000 and 2000 bp, 12,061 unigene lengths were between 2000 and 3000 bp, and 6352 unigene lengths exceeded 3000 bp. To obtain comprehensive gene function annotations, BLASTx searches were performed in the seven databases described in Section 2.2.3 (Table 3). The results identified that 67.38% of the unigenes were annotated in at least one database, with the number of gene fragments successfully annotated in the GO database being 49,311 (51.43%) compared to 16,995 (17.72%) in the KEGG database.

**Figure 1:**
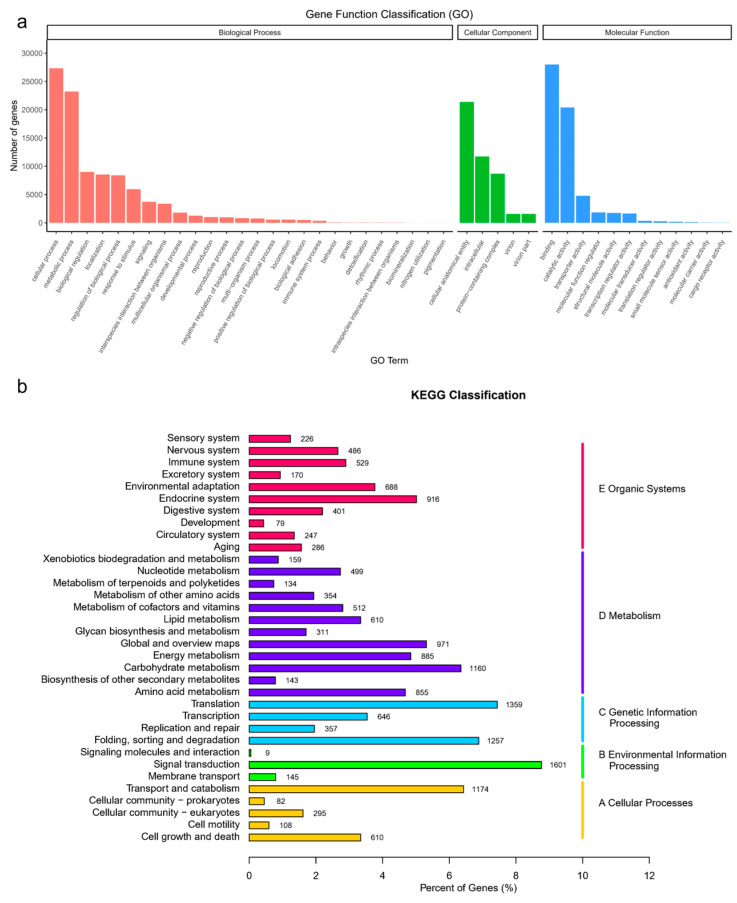
(**a**) Gene Ontology (GO) assignment and (**b**) KEGG assignment of assembled unigenes of *H. bohaiensis*.

The results obtained after searching and comparing in the GO database are shown in Figure 1a. The functions of the unigenes of *H. bohaiensis* are mainly involved in the three categories of biological process (BP), cellular component (CC), and molecular function (MF), and they are significantly enriched regarding the cellular process, metabolic process, binding, catalytic activity, cellular anatomical entity, protein-containing complex, and intracellular functions. The unigenes in the transcriptome of *H. bohaiensis* annotated by the KEGG database were classified into five branches according to the KEGG metabolic pathways (Figure 1b): (A) cellular processes, (B) environmental information processing, (C) genetic information processing, (D) metabolism, and (E) organic systems. The metabolic pathways that are more enriched are signal transduction, translation, folding, sorting and degradation, transport and catabolism, and so on.

### 3.2. Differential Gene Analysis of H. bohaiensis Under Different Nitrogen Concentration Conditions

**Figure 2:**
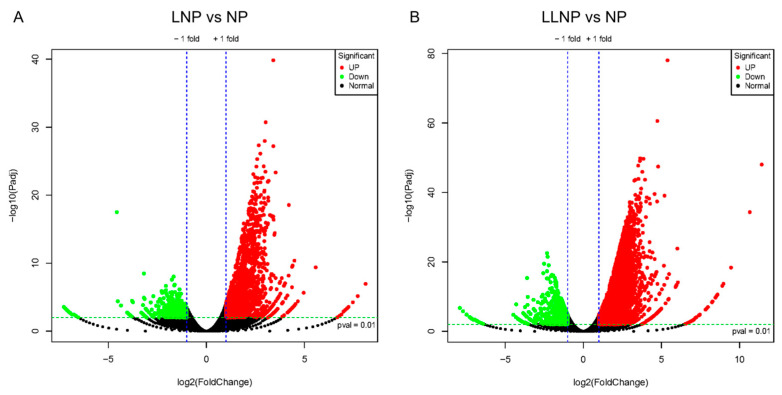
Differential genetic volcanoes of *H. bohaiensis* under different nitrogen concentration conditions ((**A**) LNP vs. NP; (**B**) LLNP vs. NP).

**Figure 3:**
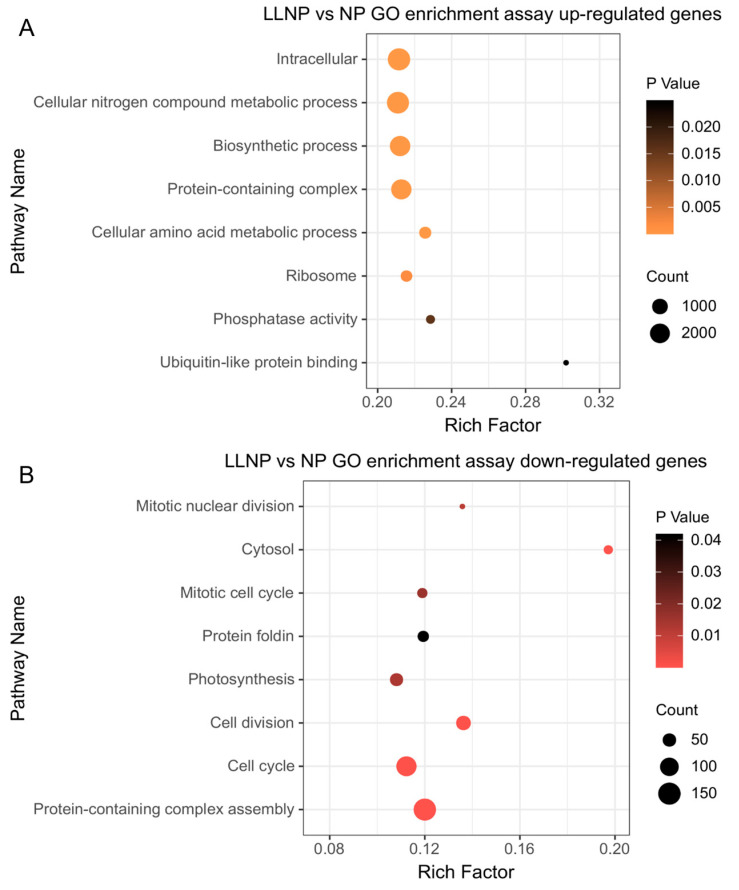
GO enrichment of differential genes under nitrogen-limited conditions in *H. bohaiensis*: (**A**) upregulated; (**B**) downregulated.

This experiment established three nitrogen concentration gradients to investigate the molecular response of *H. bohaiensis*. The differences in gene expression between the LNP, LLNP, and NP groups are illustrated in Figure 2. The control (NP) was compared to the LNP treatment and LLNP treatment separately. The number of differential genes unique to the LNP treatment was 12,333, of which 9143 genes were upregulated and 3190 genes were downregulated, and the number of differential genes unique to the LLNP treatment was 22,269, of which 15,647 genes were upregulated and 6622 genes were downregulated. The extensive transcriptional changes under LLNP conditions suggest a heightened need for cellular restructuring to adapt to compounded stress. This could lead to slower growth rates and shifts in cellular resource allocation toward survival (more upregulated genes) rather than proliferation. In natural environments, such responses might increase the competitive resilience of species like *H. bohaiensis* under limited-nutrient conditions, potentially influencing bloom dynamics. Since the LLNP has more unique differential genes compared to the LNP than the control (NP), GO enrichment was performed on the transcripts of *H. bohaiensis* cultured under LLNP conditions and compared with the NP, and the results are shown in Figure 3. The main pathways enriched for upregulation (*p* < 0.05) were cellular nitrogen compound metabolic process (GO:0034641), biosynthetic process (GO:0009058), lipid metabolic process (GO:0006629), phosphatase activity (GO:0016791), and ubiquitin-like protein binding (GO:0032182) (Figure 3A). The main pathways enriched for downregulation (*p* < 0.05) were protein-containing complex assembly (GO:0065003), cytosol (GO:0005829), cell division (GO:0051301), cell cycle (GO:0007049), mitotic nuclear division (GO:0140014), and photosynthesis (GO:0015979) (Figure 3B).

### 3.3. Differential Gene Analysis of H. bohaiensis Under Different Phosphorus Concentration Conditions

**Figure 4:**
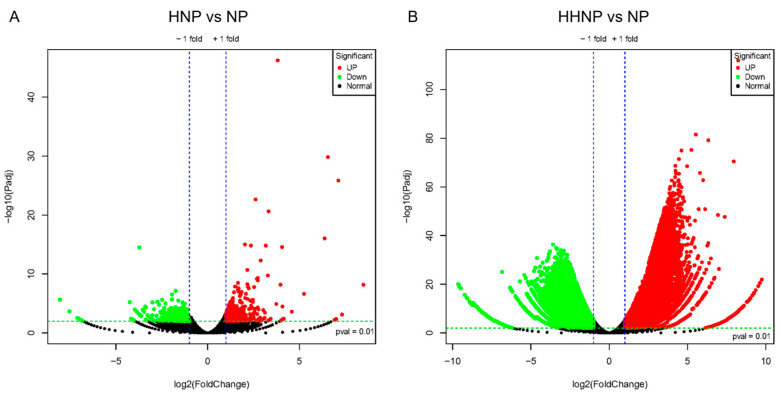
Differential genetic volcanoes of *H. bohaiensis* under different phosphorus concentration conditions ((**A**) HNP vs. NP; (**B**) HHNP vs. NP).

**Figure 5:**
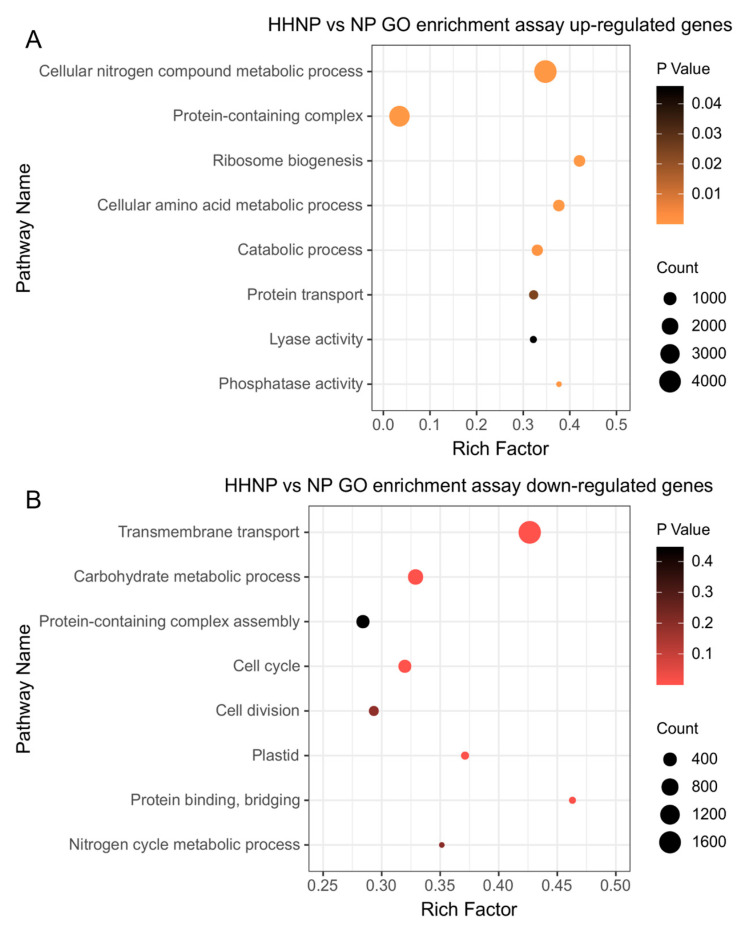
GO enrichment of differential genes under phosphorus-limited conditions in *H. bohaiensis* ((**A**) upregulated; (**B**) downregulated).

This experiment established three phosphorus concentration gradients; the control (NP) was compared to the HNP treatment and HHNP treatment separately (Figure 4). The number of differential genes unique to the HNP was 8811, of which 4603 genes were upregulated and 4208 genes were downregulated, with the lowest number of differentially expressed genes among the four nitrogen and phosphorus limitation experimental groups. The number of differential genes unique to the HHNP was 47,377, of which 24,975 genes were upregulated and 22,402 genes were downregulated; under low-phosphorus conditions (HHNP), the molecular response of *H. bohaiensis* was significant. Cells upregulate genes involved in phosphorus acquisition, such as phosphatases and phosphate transporters, to scavenge and utilize inorganic and organic phosphorus from the environment more efficiently. GO enrichment was performed on the transcripts of *H. bohaiensis* cultured under HHNP conditions and compared with the NP, and the results are shown in Figure 5. The main pathways enriched for upregulation (*p* < 0.05) were phosphatase activity (GO:0016791), protein transport (GO:0015031), catabolic process (GO:0009056), and lyase activity (GO:0016829l) (Figure 5A). The main pathways enriched for downregulation (*p* < 0.05) were carbohydrate metabolic process (GO:0005975), cell cycle (GO:0007049), plastid (GO:0009536), and cell division (GO:0051301) (Figure 5B).

### 3.4. Effects of Different Nitrogen and Phosphorus Conditions on Photosynthesis of H. bohaiensis

**Figure 6:**
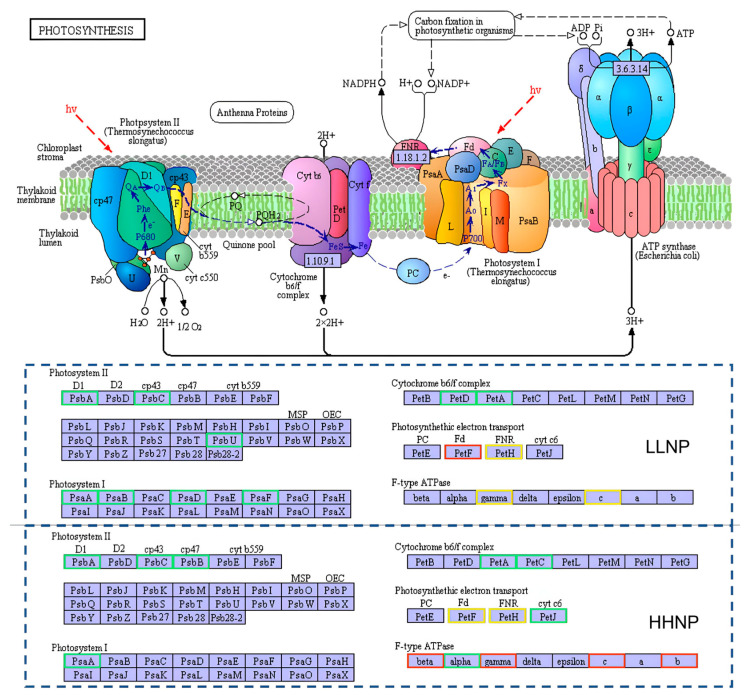
Different nitrogen (LLNP) and phosphorus (HHNP) conditions on photosynthesis enrichment pathway of *H. bohaiensis*. The red and green squares in the figure represent gene expression that is either up- or downregulated. The yellow squares represent gene expression that exhibits both up- and downregulation.

The photosynthetic pathway of *H. bohaiensis* is illustrated in Figure 6. Photosynthesis in algal cells requires the coordinated function of four components: Photosystem I (PSI), Photosystem II (PSII), the cytochrome b6/f complex, and ATP synthase. A reduction in the function of any of these components leads to a decline in microalgal photosynthesis. In the comparison between the LLNP treatment and NP treatment, significant downregulation of the differential genes was observed in three of the four components, with the downregulation factors ranging from 1.87 to 2.41, except for the ATP synthase gene. In the comparison between the HHNP treatment and NP treatment, four genes (F-type H^+^/Na^+^-transporting ATPase subunit β, F-type H^+^-transporting ATPase subunit γ, F-type H^+^-transporting ATPase subunit c, and F-type H^+^-transporting ATPase subunit b) related to ATP synthase were found to be upregulated, with fold changes ranging from 0.84 to 5.99, while the other three components also exhibited significant downregulation of the differential genes, with downregulation factors between 2.03 and 3.10. Downregulated photosynthesis limits the energy available for carbon fixation and biomass accumulation, leading to reduced growth rates. This could slow the onset of blooms in *H. bohaiensis* under nutrient-depleted conditions and affect the competition with other phytoplankton.

### 3.5. Effects of Different Nitrogen and Phosphorus Conditions on Carbon Fixation in Photosynthetic Organisms of H. bohaiensis

**Figure 7:**
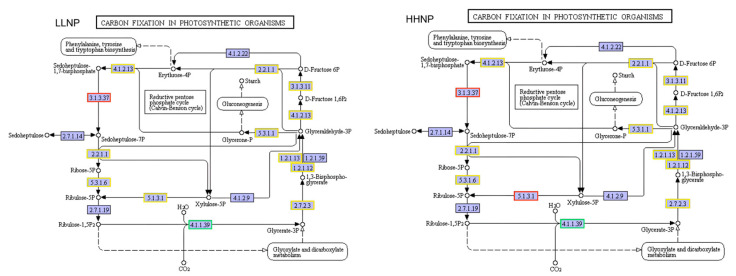
Different nitrogen (LLNP) and phosphorus (HHNP) conditions on carbon fixation in photosynthetic organism enrichment pathway of *H. bohaiensis.* The red and green squares in the figure represent gene expression that is either up- or downregulated. The yellow squares represent gene expression that exhibits both up- and downregulation.

The carbon fixation in the photosynthetic organism pathway of *H. bohaiensis* is illustrated in Figure 7. The carbon fixation process in microalgal cells primarily occurs through the utilization of the Calvin cycle during photosynthesis. The comparative analysis between the LLNP treatment and NP treatment showed a significant upregulation of the gene expression encoding sedoheptulose-1,7-bisphosphatase (SBPase, EC 3.1.3.37), with a 1.99-fold increase. On the other hand, the gene expression of three genes encoding ribulose-bisphosphate carboxylase (RuBisCO, EC 4.1.1.39) was observed to be downregulated, with fold changes ranging from 1.02 to 1.16. In the comparison between the HHNP treatment and NP treatment, the gene expression encoding SBPase showed a remarkable upregulation of 3.05-fold, while the gene expression of the three genes encoding RuBisCO was found to be downregulated, with fold changes ranging from 1.53 to 1.97. Additionally, the gene expression of the two genes encoding ribulose-phosphate 3-epimerase (RPE, EC:5.1.3.1) was significantly upregulated, with fold changes of 3.39 and 4.21, respectively.

### 3.6. Effects of Different Nitrogen and Phosphorus Conditions on the Proteasome of H. bohaiensis

**Figure 8:**
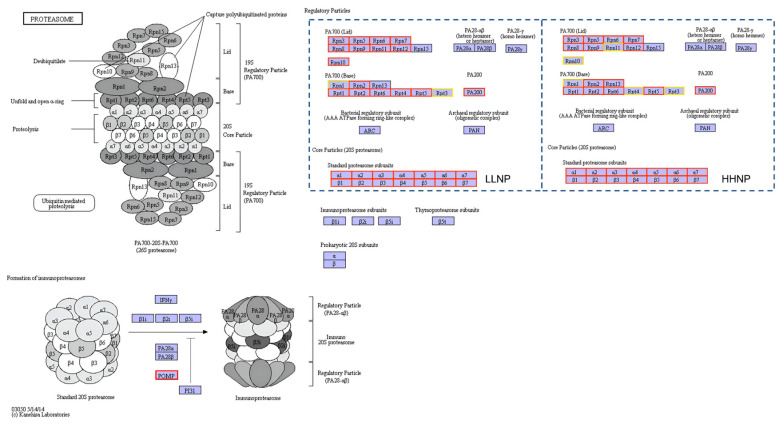
Different nitrogen (LLNP) and phosphorus (HHNP) conditions on proteasome enrichment pathway of *H. bohaiensis*. The red squares in the figure represent gene expression that is upregulated. The yellow squares represent gene expression that exhibits both up- and downregulation.

The enrichment pathways of the proteasome in *H. bohaiensis* are depicted in Figure 8. Under nutrient limitation, the proteasome pathways in *H. bohaiensis* were consistently upregulated, highlighting the organism’s adaptive response to environmental stressors. Specifically, the proteasome pathway in the LLNP treatment was significantly upregulated compared to the NP treatment. All the genes significantly expressed in this pathway were upregulated, with no downregulated genes observed. The genes encoding 14 subunits of the 20S proteasome were upregulated by factors ranging from 1.4 to 2.76, while the genes for 11 subunits of the 26S proteasome regulatory complex showed upregulation by factors between 1.25 and 2.47. Similarly, the proteasome pathway in the HHNP treatment also exhibited significant upregulation compared to the NP treatment. Among the genes significantly expressed in this pathway, the genes for 14 subunits of the 20S proteasome were upregulated by factors ranging from 1.09 to 2.42. Furthermore, three additional upregulated genes for the 26S proteasome regulatory complex were identified in the HHNP treatment, with upregulation factors between 1.26 and 3.38. The upregulation of the proteasome pathways under nutrient-limiting conditions reflects the critical role of protein degradation in nutrient recycling. By breaking down damaged or unneeded proteins, *H. bohaiensis* can liberate amino acids and other essential metabolites, which can then be recycled for new protein synthesis and energy production. This process not only facilitates nutrient conservation but also enhances cellular stress adaptation, enabling the organism to maintain homeostasis and survive under unfavorable conditions.

### 3.7. Effects of Different Nitrogen and Phosphorus Conditions on Nitrogen Metabolism in H. bohaiensis

**Figure 9:**
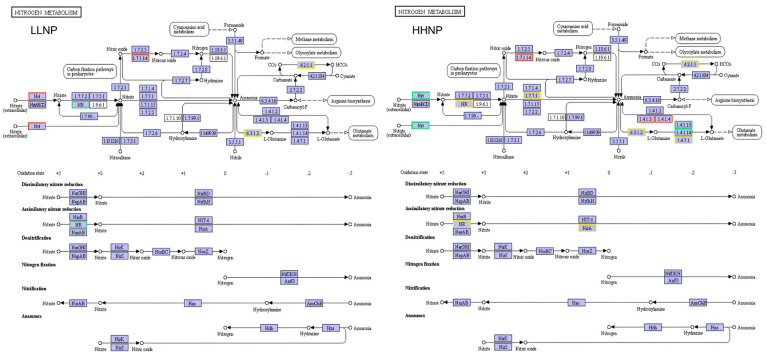
Different nitrogen (LLNP) and phosphorus (HHNP) conditions on nitrogen metabolism enrichment pathway of *H. bohaiensis*. The red and green squares in the figure represent gene expression that is either up- or downregulated. The yellow squares represent gene expression that exhibits both up- and downregulation.

The nitrogen metabolism pathway of *H. bohaiensis* is shown in Figure 9. Nitrogen metabolism comprises six primary pathways: assimilatory nitrate reduction, assimilatory nitrate assimilation, denitrification, nitrogen fixation, nitrification, and anammox.

In the comparison between the LLNP treatment and the NP treatment, the genes encoding the proteins involved in nitrogen uptake, such as transport proteins Nrt, nitric oxide reductase (EC 1.7.1.14), and glutamine synthetase (GS), were detected as positively regulated. Nrt showed an upregulation factor of 3.54; nitric oxide reductase was upregulated by 2.70; among the seven GS genes, five were upregulated, with factors ranging from 1.91 to 7.49, while two were downregulated by factors of 2.08 and 2.40, respectively.

In the comparison between the HHNP treatment and the NP treatment, the genes encoding the proteins involved in nitrogen uptake, including glutamine synthetase (GS), glutamate synthase (GOGAT), glutamate dehydrogenase (GDH), nitrate reductase (NR), and nitrite reductase (NiR), were also found to be positively regulated. NR and NiR may be involved in the reduction of nitrate and nitrite to ammonium, respectively. In the dataset, two NR genes were upregulated by 6.08 and 8.92 times, while two NR genes were downregulated by 2.26 and 2.90 times. One NiR gene was downregulated by 2.19 times, and another by 4.14 times. Among the thirty-six GS-related genes, six genes were upregulated by factors ranging from 1.48 to 1.92 under phosphorus limitation, while the remaining genes were downregulated, although their expression levels were relatively low. One GOGAT gene was upregulated by 6.74 times, while four GOGAT genes were downregulated by factors ranging from 2.03 to 2.60. Under phosphorus limitation, the Nrt gene was downregulated by 3.27 times, whereas a type of ammonium assimilating enzyme, GDH, was upregulated by 4.62 times. Intracellular nitrite can directly interact with ferredoxin-nitrite reductase (EC 1.7.7.1) to be converted into ammonia, with one gene showing upregulation by 2.19 times; alternatively, it can first be converted to nitrogen through the action of nitric oxide reductase (EC 1.7.2.5/EC 1.7.1.14).

## 4. Discussion

The GC content of the five experimental sequencing samples ranged from 60% to 65%, with all the samples exhibiting Q30 base percentages exceeding 92%, indicating high sequencing quality suitable for analysis. At the time of sampling on day 27, *H. bohaiensis* was found to be under nitrogen limitation in the LLNP treatment and phosphorus limitation in the HHNP treatment. Both the nitrogen and phosphorus limitations evoked molecular responses in *H. bohaiensis*, albeit with notable differences. The volcano plot analyses indicated that greater divergence from the control (NP) in nutrient conditions corresponded with more pronounced gene expression differences. Consequently, this study focused on analyzing the two treatments (LLNP and HHNP) that exhibited the most significant differential gene expression compared to the control. The study revealed distinct responses under nitrogen and phosphorus limitations. Managing the nutrient ratios to favor conditions less conducive to HABs could suppress the dominance of *H. bohaiensis*. Aquaculture ponds often serve as nutrient hotspots, particularly for nitrogen and phosphorus. By explicitly connecting the findings to ecosystem and aquaculture management, it is possible to develop targeted strategies to mitigate HABs, ensuring both environmental sustainability and economic viability in aquaculture industries.

Under nitrogen and phosphorus limitations, the GO enrichment analysis revealed downregulation of those functions associated with cell division and the cell cycle, suggesting that these limitations inhibit the cell division process in *H. bohaiensis*, resulting in slower cellular activity. Prolonged cell division cycles could extend the generation time of the microalgae, leading to reduced division rates and, consequently, lower algal density. This deceleration in cell division may represent a strategy employed by microalgae to cope with external nutrient limitations [26].

The KEGG enrichment analysis of the photosynthetic pathways in *H. bohaiensis* revealed significant downregulation of these pathways under nitrogen and phosphorus limitations. This observation was consistent with previous findings [27] that showed a reduction in photosynthetic pigment content under similar stress conditions. Nitrogen was a critical element for chlorophyll synthesis; therefore, under nitrogen limitation, the availability of nitrogen for chlorophyll production decreased, which may explain the observed reduction in the chlorophyll levels within the algal cells. In the phosphorus-limited conditions, although there was a decrease in the intracellular photosynthetic pigments, an upregulation of the ATP synthase genes occurred. This adaptation likely served to enhance the ATP production, thereby providing additional energy to the cells facing phosphorus stress [28]. The enhanced ATP synthesis was pivotal for sustaining the essential cellular processes and mitigating the detrimental impacts of nutrient scarcity. The upregulation of ATP synthase enabled the cells to better sustain their metabolic processes, facilitate energy-intensive responses to stress, and ultimately enhance their resilience in challenging environmental conditions. The KEGG enrichment analysis of the proteasome pathways in *H. bohaiensis* under nitrogen and phosphorus limitations revealed a significant upregulation of these pathways. The primary function of the proteasome was to degrade unnecessary or damaged cellular proteins, indicating that *H. bohaiensis* could break down intracellular proteins under both nitrogen- and phosphorus stress conditions. Previous studies [27] indicated that *H. bohaiensis* could store nitrogen sources intracellularly during nutrient limitation, enabling it to sustain growth over extended periods. Additionally, as *H. bohaiensis* grew, the intracellular protein content gradually decreased, likely due to proteasome-mediated degradation. The significant upregulation of the proteasome pathways might have represented one of the molecular mechanisms by which *H. bohaiensis* utilized its intracellular nitrogen reserves to survive in nitrogen-deficient environments. Research on *Prorocentrum donghaiense* demonstrated that nitrogen starvation led to a reduction in the cellular protein content [29]. When the cells encountered a nutrient limitation, the proteins could serve as an organic nitrogen reservoir [30]. The cellular proteins contained substantial nitrogen stores; however, due to size constraints and the stability of the proteins associated with organic minerals, they could not be directly absorbed by the algal cells. Therefore, the cells had to first hydrolyze these high-molecular-weight organic nitrogen compounds into smaller soluble molecules, such as amino acids, to facilitate nitrogen recycling within the cell.

Under nitrogen-limiting conditions, the genes encoding nitrate transporters (Nrt) and nitric oxide reductases were upregulated, suggesting that *H. bohaiensis* might have increased the transport of various substances by enhancing the transporter proteins and also sought to obtain additional nitrogen sources through the reduction of nitric oxide during nitrogen limitation. In phosphorus-limiting conditions, a notable downregulation of the nitrogen metabolism pathways occurred, which aligned with previous experimental findings [27]. The transport of nitrate into the cell required adenosine triphosphate (ATP), and the phosphate concentration affected ATP synthesis [31]. Consequently, the activity of the algal nitrate reductases was constrained by the concentration of PO₄³⁻. Within the nitrogen metabolism pathway, the gene for the nitrate transporter was downregulated by 2.27-fold, which explained the lower nitrate uptake rates observed in *H. bohaiensis* during the later growth stages. Intracellular nitrite could be directly converted to ammonia by nitrite reductase (EC 1.7.7.1) through its interaction with ferredoxin. Alternatively, it could be converted into nitrogen via nitrite reductase (EC 1.7.2.1) and nitric oxide reductase (EC 1.7.2.5/EC 1.7.1.14) and subsequently transformed into ammonia through the action of nitrogenase (EC 1.18.6.1/EC 1.19.6.1) and other reductases (EC 1.7.2.4). The upregulation of the genes encoding these enzymes likely represented a molecular response strategy employed by *H. bohaiensis* under growth-limiting conditions. Ultimately, these findings suggest that enhanced nitrogen uptake mechanisms may provide competitive advantages to *H. bohaiensis*, particularly in environments where nitrogen availability is critical for bloom dynamics and survival strategies. Similarly, *P. donghaiense* optimized the utilization of environmental nitrogen sources under nutrient-limiting conditions by upregulating the expression of those genes related to nitrate and ammonium transporters [32].

## 5. Conclusions

This study investigated the transcriptome of *H. bohaiensis* under varying nutrient conditions, with a focus on differential gene expression. The results showed that, the greater the nutrient deviation from the control conditions, the more differentially expressed genes were identified. Under nutrient-limited conditions, the biosynthesis, catabolic processes, and phosphatase activity were upregulated, while the cell cycle and cell division processes were downregulated. The KEGG analysis showed that nitrogen limitation upregulated the proteasome-related pathways while downregulating photosynthesis and carbon fixation significantly. Under low-phosphorus conditions, the proteasome pathways were also upregulated, with nitrogen metabolism significantly downregulated. These findings suggest that *H. bohaiensis* adapts to nutrient stress by modulating its metabolic processes to maintain growth.

## Figures and Tables

**Table 1:** Initial NO^3−^ and PO_4_^3−^ concentrations in the medium for *H. bohainensis*.

Treatments	NO^3−^ (μM)	PO_4_^3−^ (μM)	N:P Ratios
NP (control)	880	32	27.5
LNP	512	32	16
LLNP	128	32	4
HNP	880	16	55
HHNP	880	8	110

**Table 2:** Overview of *H. bohaiensis* transcriptome data.

Treatments	Raw Reads	Clean Reads	Q30 Percentage (%)	GC Content (%)
NP	23,642,227	21,903,878	93.24	64.40
LNP	22,865,692	20,966,940	95.01	63.88
LLNP	23,701,660	22,209,499	92.72	62.28
HNP	22,738,505	22,408,956	93.32	63.84
HHNP	23,997,247	22,114,352	94.45	60.03

**Table 3:** Annotation results of transcriptome genes in various databases.

Database	The Number of Annotated Genes	BLASTx Analysis Percentage (%)
NR	52,180	54.43
GO	49,311	51.43
KEGG	16,995	17.72
NT	4415	4.6
Swiss-Prot	30,542	31.86
Pfam	49,312	51.44
KOG	16,047	16.73

## Data Availability

The original contributions presented in this study are included in the article/Supplementary Material. Further inquiries can be directed to the corresponding author.

## Supplementary Materials

The following supporting information can be downloaded at https://www.mdpi.com/article/10.3390/microorganisms12122665/s1, Figure S1: Growth curves of *H. bohaiensis* in varied nutrient conditions.
