# Supplementary figures and images for: Transcriptome Analysis of the Harmful Dinoflagellate Heterocapsa bohaiensis Under Varied Nutrient Stress Conditions
Source: Microorganisms. 2024 Dec 22;12(12):2665. doi: 10.3390/microorganisms12122665 (PMC11728646; doi:10.3390/microorganisms12122665)

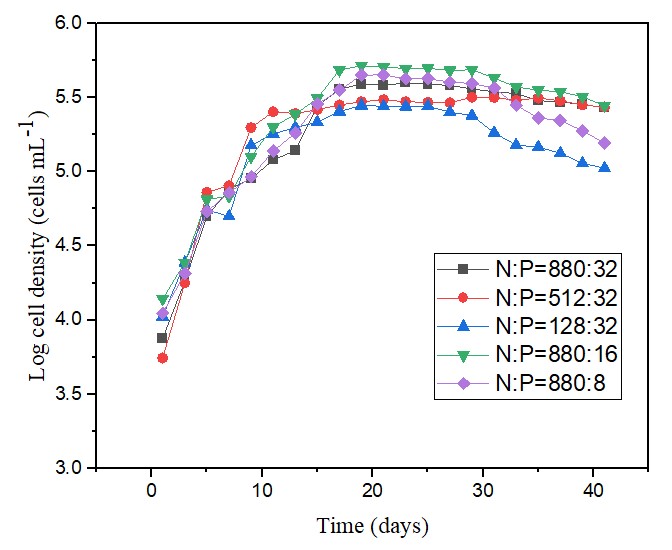

Supplement: Supplementary file 1 [file microorganisms-12-02665-s001.zip › microorganisms-3335039-supplementary.jpg]
